# Synthesis of Carbon Nanotubes in Thermal Plasma Reactor at Atmospheric Pressure

**DOI:** 10.3390/nano7020045

**Published:** 2017-02-18

**Authors:** Lukasz Szymanski, Zbigniew Kolacinski, Slawomir Wiak, Grzegorz Raniszewski, Lukasz Pietrzak

**Affiliations:** Institute of Mechatronics and Information Systems, Lodz University of Technology, 90-924 Lodz, Poland; zbigniew.kolacinski@p.lodz.pl (Z.K.); slawomir.wiak@p.lodz.pl (S.W.); grzegorz.raniszewski@p.lodz.pl (G.R.); lukasz.pietrzak@p.lodz.pl (L.P.)

**Keywords:** carbon nanotubes, plasma reactor, plasma synthesis, ferromagnetic carbon nanotubes

## Abstract

In this paper, a novel approach to the synthesis of the carbon nanotubes (CNTs) in reactors operating at atmospheric pressure is presented. Based on the literature and our own research results, the most effective methods of CNT synthesis are investigated. Then, careful selection of reagents for the synthesis process is shown. Thanks to the performed calculations, an optimum composition of gases and the temperature for successful CNT synthesis in the CVD (chemical vapor deposition) process can be chosen. The results, having practical significance, may lead to an improvement of nanomaterials synthesis technology. The study can be used to produce CNTs for electrical and electronic equipment (i.e., supercapacitors or cooling radiators). There is also a possibility of using them in medicine for cancer diagnostics and therapy.

## 1. Introduction

The process of the continuous synthesis of carbon nanotubes (CNTs) on metallic surfaces is of special interest for many technological applications. The classic synthesis methods reviewed in Section 2 and Section 3 have not been developed enough before to be used in a continuous way. The synthesis of carbon nanotubes in thermal plasma seems to better fulfill the above requirement.

The main objective of this work was to develop a new system for continuous processing of carbon nanotube deposition on a current-conductive surface using thermal plasma. In order to ensure plasma stability and easy control, the microwave plasma jet was selected. As a substrate, a thin metal strip was used, i.e., as a mega-active electrode surface required in the next generation of electrolytic batteries and supercapacitors can be chosen. 

## 2. CNT Synthesis Methods

General carbon nanotubes can be produced using many simple processes as well as very sophisticated ones. In the simple carbon arc methods, synthesis takes place in reactors working at a pressure lower than the atmospheric one [1]. Carbon nanotubes are produced in an electric arc between two graphite electrodes [2,3,4,5]. The electric arc reactors with magnetic stabilization of the arc voltage have a similar design structure [6,7]. In the most simplified modifications of this method, reactors for the synthesis of CNTs use an AC power supply [8,9,10].

In the laser synthesis methods of carbon nanotubes, called laser chemical vapor deposition (LCVD), a graphite target is under continuous evaporation by a high-temperature laser beam [11,12,13,14,15,16,17,18,19,20]. Solar radiation and electrolytic methods can also be used for CNT synthesis in reactors working at atmospheric pressure [21,22,23,24,25,26,27,28,29,30,31,32].

In the sonochemical methods, carbon nanotubes are obtained in a homogeneous process with an ultrasonic generator (600 W, 20 kHz) [33]. Another method of carbon nanotube synthesis is growing them on a matrix made of a catalytic AAO (anodic aluminum oxide). CNTs can also be produced by the carbonization (evaporation) of the emulsion copolymers of polyacrylonitrile [34,35]. Another set of methods that should be mentioned are plasma-assisted methods (plasma-enhanced chemical vapor deposition—PECVD) [36,37,38,39]. Some important design upgrades related to those methods are presented in this paper.

## 3. CVD Reactors for the Synthesis of CNTs from the Gas Phase Working at Atmospheric Pressure

**Figure 1:**
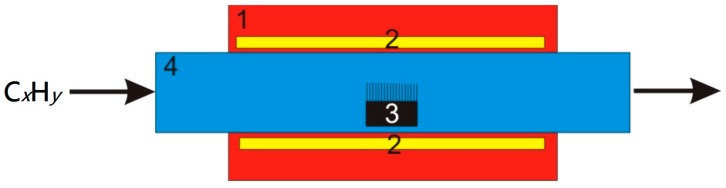
Setup of CVD reactor for CNT synthesis where: 1—furnace; 2—furnace heating elements; 3—substrate with growing nanotubes; 4—reaction tube [41].

This paper focuses on reactors for the CNT synthesis operating at atmospheric pressure. In this case, the most common method is the CVD method. In the CVD reactor, a decomposition of fluid- or carbon gas–containing substances takes place. Then, the catalyst helps to form atoms in hexagonal configurations of the grapheme-like wall of carbon nanotubes. The gases acetylene, benzene, ethylene, methane, propylene or carbon monoxide can be applied as the carbon sources. The catalysts are iron, cobalt, nickel and other metals. There are also reactors, in which there is no need to use a catalyst, even for the production of Single-Walled Carbon Nanotubes (SWNTs). CNT synthesis takes place in the flow system of a noble gas in the temperature range from 800 K to 1000 K for Single Multi-Walled Carbon Nanotubes (MWCNTs) and from 1100 to 1250 K for SWNTs. However, in this method some difficulties appear with the activation of the catalyst being initially in the liquid or solid state. The use of injection or steaming containers (boats) in a horizontal furnace does not guarantee that the process will keep the stable parameters to remain continuous [40,41,42,43,44,45,46,47,48]. The general setup of the reactor is shown in Figure 1.

## 4. Experimental Setup for CNT Plasma Synthesis 

**Figure 2:**
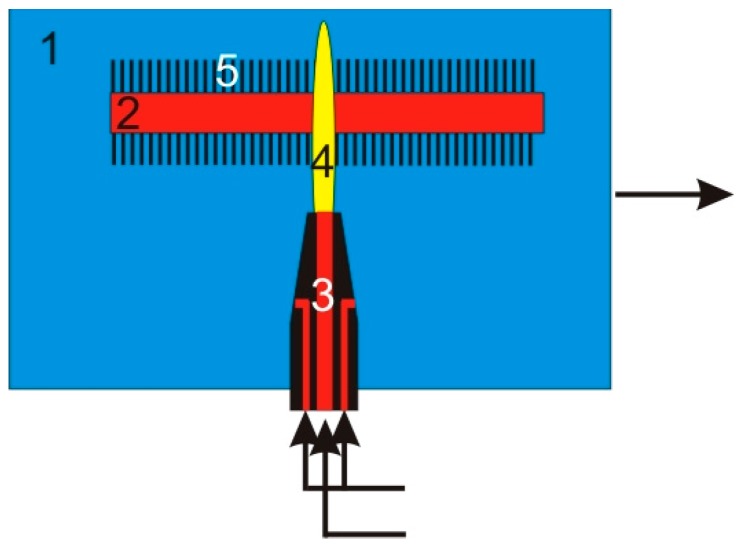
The general setup of the microwave plasma reactor where: 1—reaction chamber; 2—substrate with catalyst layer; 3—plasma nozzle; 4—plasma; 5—carbon nanotubes.

**Figure 3:**
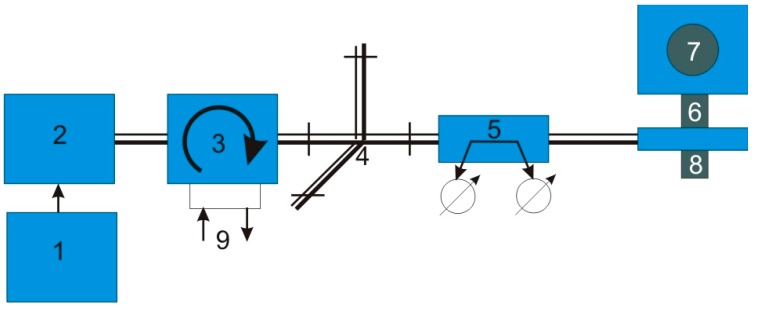
Block diagram of a test line where: 1—power supply; 2—generator; 3—circulator; 4—matching circuit, 5—reflectometer; 6—coaxial plasma head; 7—reaction chamber; 8—gas inlets; 9—cooling water.

**Figure 4:**
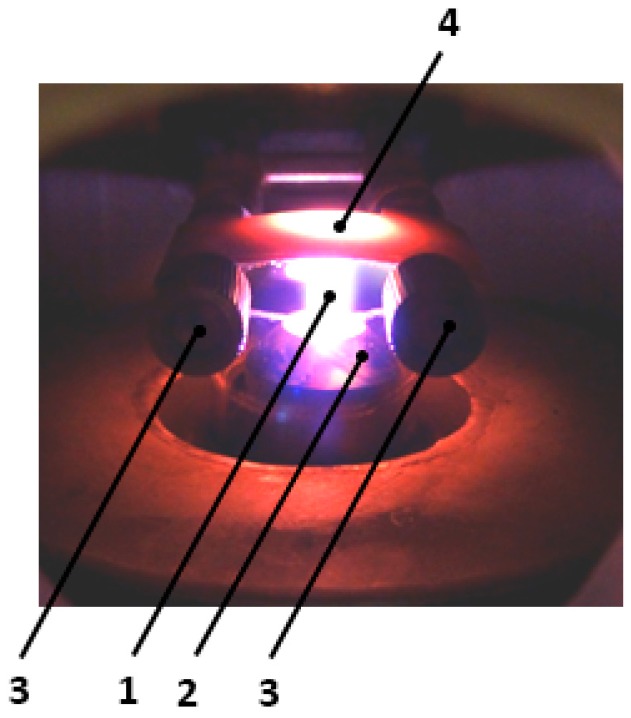
Photo of reaction zone of the microwave plasma reactor for carbon nanotube (CNT) synthesis on a metal substrate where: 1—microwave plasma; 2—plasma head; 3—substrate feeder; 4—substrate.

In our laboratory, we built a reactor that allows the synthesis of carbon nanotubes on substrates with the application of microwave plasma. This reactor uses only one source of energy for simultaneously heating the substrate and breaking the bounds of the reaction gases. The substrate for the synthesis was made of stainless steel (1.4310) with thicknesses of 0.005, 0.01, 0.02, 0.05 mm, respectively. The substrate temperature (1000–1300 K) was controlled by the pyrometer. Its electric output signal was used in the loop for temperature control by tuning the microwave plasma power supply (max. 800 W). A mixture of argon and nitrogen was supplied axially to the microwave plasma nozzle. A mixture of argon, nitrogen, hydrogen and ethylene was introduced orthogonally to the plasma jet. The reactor setup is shown in Figure 2, a block diagram of the processing line in Figure 3 and a photo of the reactor in Figure 4.

## 5. Thermal Decomposition of Gaseous Reagents

**Figure 5:**
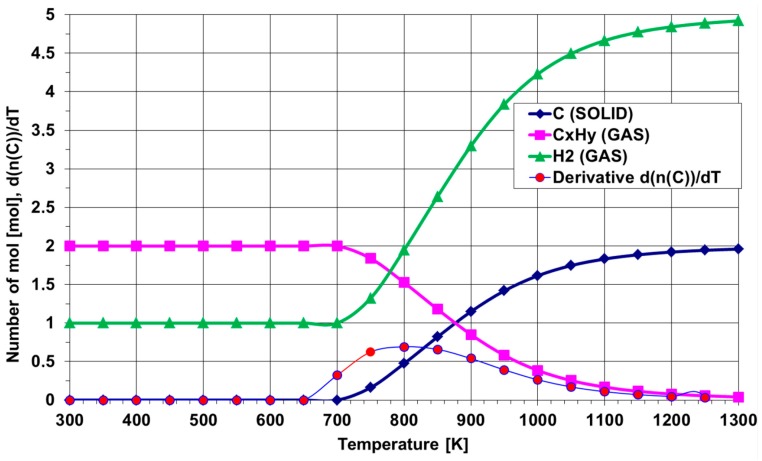
Thermal decomposition of the mixture of 1000 sccm of ethylene with 3000 sccm of hydrogen.

The synthesis process is composed of several stages such as venting, substrate annealing, and chemical reduction with hydrogen. Every step has its own range of allowed parameters (gas flow, time and temperature). At the beginning of the work, some computer simulations were conducted. During these calculations, the flow ranges of hydrogen, ethylene, acetylene, methane and other gaseous reagents were considered. Figure 5 shows the thermal decomposition of the mixture of two gases: 1000 sccm of ethylene and 3000 sccm of hydrogen. This proportion was commonly used in our research. It was concluded that from a thermodynamic point of view, the temperature for which there is a maximum of the first derivative of d(*n*(C))/dT shows the optimal point in which the separation of solid carbon in the reaction of the spontaneous decomposition of hydrocarbons is expected.

The temperature of 800 K is the minimum value to obtain carbon in a solid stage that is suitable for synthesis of carbon nanotubes. 

Completed simulations are consistent with the data presented in the literature. For the purpose of comparison, Table 1 presents a review of the synthesis conditions found in the literature.

## 6. Experimental Setup for Continuous CNT Synthesis 

**Figure 6:**
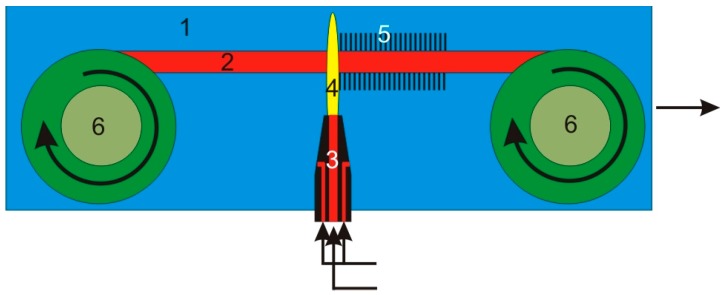
Schematic diagram of the continuous microwave plasma system for CNT synthesis where: 1—reactor space; 2—substrate-metal strip; 3—microwave plasma head; 4—microwave plasma torch; 5—CNTs; 6—cassettes to move the metal substrate.

The reactor described in Section 4 can receive single samples only. The design presented below is an upgrade of the previous design which allows continuous movement of the metal substrate. The general principles of the reactor working are presented in Figure 6. 

**Figure 7:**
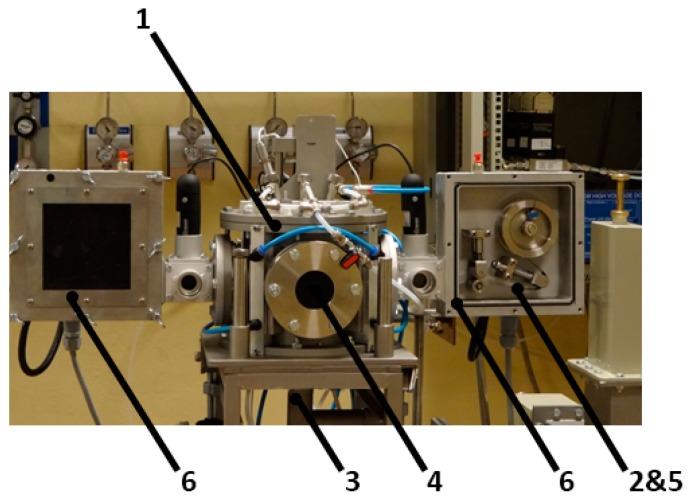
Photo of the plasma processing system where: 1—reactor chamber; 2—substrate-metal strip; 3—microwave plasma head; 4—microwave plasma torch (not visible); 5—CNTs (not visible); 6—cassettes to move the metal substrate.

In Figure 7, the photo of the real system is shown. The synthesis of carbon nanotubes took place in the chamber on a metal strip (stainless steel) prepared by depositing a catalyst layer on the strip surface. The system has two moving tape cassettes supplying the reaction chamber with the substrate strip.

The synthesis of carbon nanotubes took place in a stainless steel chamber (purged 30 min with air plasma before growth started) on a metal substrate, which is ideal for this purpose. Argon (purity: O_2_ < 2 ppm, N_2_ < 5 ppm, H_2_O < 2 ppm, CH_4_ < 0.5 ppm, CO < 0.5 ppm, CO_2_ < 0.5 ppm) and nitrogen (purity: O_2_ < 2 ppm, H_2_O < 3 ppm) were used as the plasma gases whereas hydrogen (purity: O_2_ < 2 ppm, N_2_ < 3 ppm, H_2_O < 5 ppm, C*_x_*H*_y_* < 0.5 ppm) and ethylene (purity: O_2_ < 15 ppm, N_2_ < 50 ppm, C*_x_*H*_y_* < 450 ppm) provided carbon for the formation of CNTs. Carbon-carrying gas was supplied through a microwave plasma nozzle where it was decomposed. Plasma heated the substrate with a catalyst applied on the surface. The catalyst, ferrocene, was firstly dissolved in xylene. The surface was painted by this fluid in a dip-coating mode before the process started. The temperature of the substrate was controlled by the IR thermometer. The substrate moving speed over the plasma jet ranged from 0.01 m/h up to 0.1 m/h. Figure 7 shows the photo of the reactor.

## 7. Results 

For the synthesis of carbon nanotubes, the stainless steel 1.4310 (1.4310-X10CrNi) cold-rolled was used. Its density was 7.9 kg/dm^3^, with a specific heat of 500 J/(kgK), a thermal conductivity of 15 W/(mK), a resistivity of 0.73 (mm^2^)/m, and it consisted of 17% Cr, 7% Ni, 0.05%–0.15% C, <2.00% Si, <2.00% Mn, <0.045% P, <0.015% S, 16.00%–19.00% Cr, 6.0%–9.5% Ni, <0.80% Mo.

The following methods of making the catalyst layers on the substrate surface were used:

- chemical methods: coating the steel strip (substrate) with a layer of an antioxidant, e.g., Al_2_O_3_ applied in a dip-coating manner, followed by the application of an oxide catalyst layer such a Fe_2_O_3_ on the top,
- treatment of the substrate surface with a ferrocene catalyst. Ferrocene evaporates from the source placed inside the reactor chamber which is a currently patented method by the paper authors.

**Figure 8:**
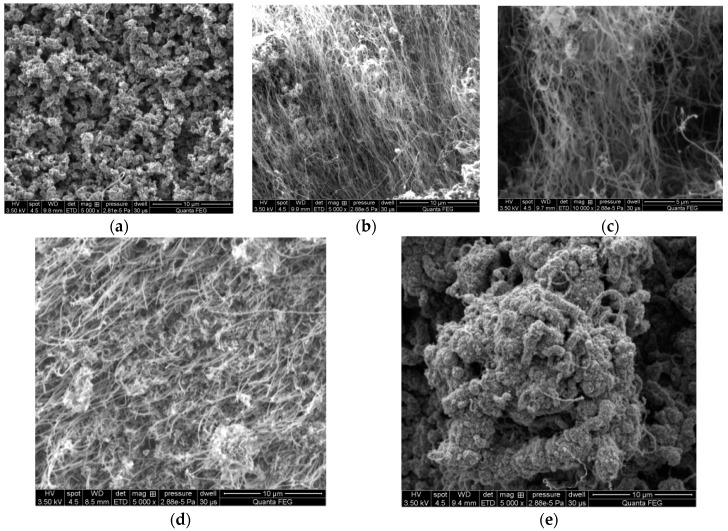
SEM images of the steel with carbon nanotubes synthesized at different temperatures (**a**) 1020 K; (**b**) 1050 K; (**c**) 1070 K; (**d**) 1100 K; (**e**) 1120 K.

One can see Scanning Electron Microscope (SEM) micrographs of deposits corresponding to different process temperatures (Figure 8a–e) within the 100 K range below. In order to present these relationships, adequate SEM images were selected, allowing observation of the process products.

The research results proved that the carbon nanotubes were synthesized on the steel surface at temperatures ranging from 1020 K to 1120 K (Figure 8a–e). Temperatures outside of described range did not allow CNT synthesis.

It could be noted that the most desirable synthesis temperature range is from 1050 K to 1070 K. Both 1050 K and 1070 K temperatures resulted in carbon nanotubes growing in the form of a “carpet” perpendicular to carpet’s base.

For chemical composition and structural examination, transmission electron microscopy (TEM—Tesla BS 512 with YAG camera, Pardubice, Czechoslovakia), energy-dispersive X-ray spectroscopy (EDX), and thermogravimetry (TGA—TA Instruments 2950 TGA HR apparatus, New Castle, DE, USA) techniques were used.

**Figure 9:**
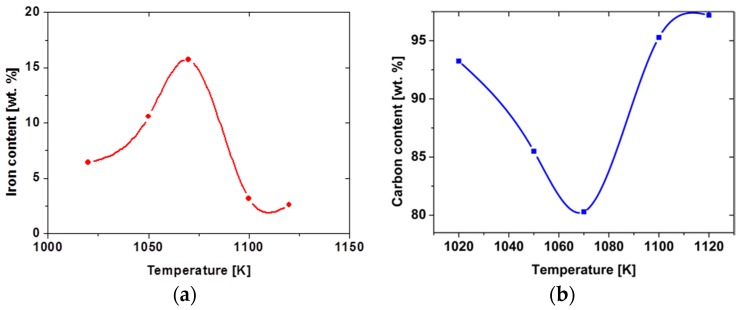
Plots of iron—(**a**) and carbon—(**b**) content versus synthesis temperature.

EDX analysis results (obtained using a FEI Quanta 200 FEG Mark II scanning electron microscope (New Castle, DE, USA) with an EDS Oxford INCA Energy System 250 spectrometer, Hillsboro, OR, USA) for CNT samples synthesized above the temperature range are shown in Figure 9a,b. Results presented on the plots are average values calculated for 10 samples synthesized under the same experimental conditions. Only Fe and C elements were analyzed, since other elements’ signals were the steel strip (CNT base) elements’ signals. 

**Figure 10:**
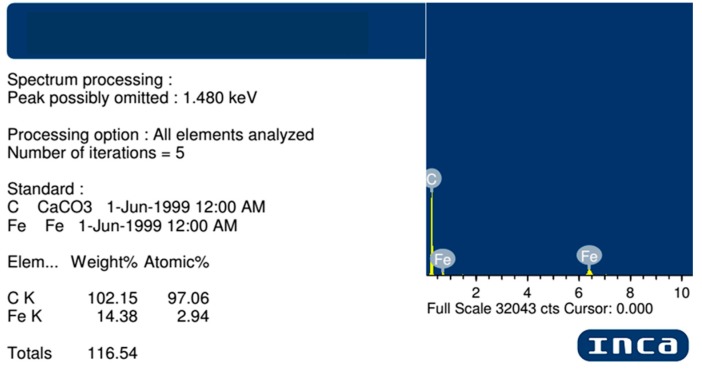
Energy-dispersive X-ray spectroscopy (EDX) analysis results for one of the specimens synthesized at 1070 K.

Significant data from EDX analysis proves a high carbon level for all samples and a high iron content, especially for the 1070 K (almost 20 wt. %) synthesis temperature sample. Figure 10 shows one of the EDS analysis results, which the average values shown on the plots in Figure 9 were calculated from. 

**Figure 11:**
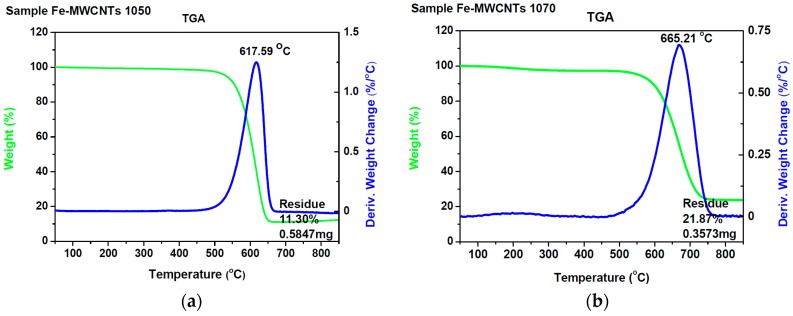
Thermogravimetry (TGA) thermographs of samples synthesized at (**a**) 1050 K; (**b**) 1070 K.

As a complement to the EDX analysis, the TGA technique was used. In Figure 11a,b, typical thermographs for 1050 K and 1070 K are shown.

**Figure 12:**
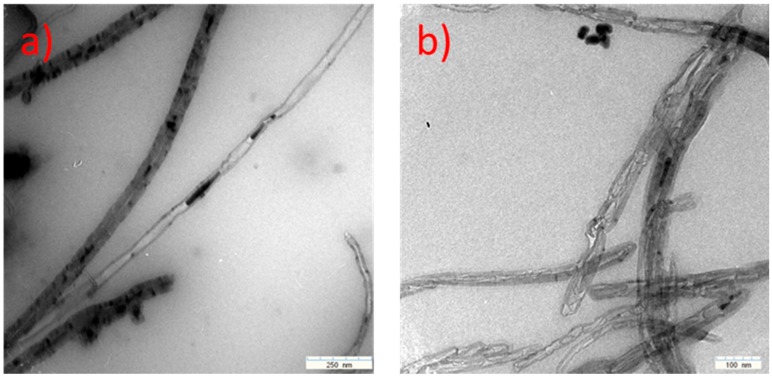
Transmission electron microscopy (TEM) images of Multi-Walled Carbon Nanotubes (MWCNTs) obtained during synthesis at (**a**) 1050 K; (**b**) 1070 K, respectively.

It may be noticed that the residue values were similar to the iron content achieved by EDX analysis. Also, maximum speed oxidation peaks for the examined samples lie in the 870–930 K temperature range and are similar to those described in the literature [56] as typical for multiwalled carbon nanotubes. The TEM examination of 1050 K and 1070 K samples revealed a multiwalled CNT structure and Fe nanoparticles encapsulated inside the MWCNTs (Figure 12).

## 8. Conclusions 

The experimental results proved that the continuous synthesis of CNTs on a metal strip using a microwave thermal plasma reactor at atmospheric pressure can be done. Using analytical and diagnostic techniques let us hypothesize that successful growth of MWCNTs took place. The presence of multiwalled carbon nanotubes in the tested material was confirmed by both TGA and TEM experimental results. TGA showed oxidation temperatures typical for MWCNTs and, also, with EDX examination, provided information about the Fe content. Fe placement in CNTs is very meaningful for future applications of the obtained product. TEM analysis showed successful Fe encapsulation, which opens the way for medical applications of nanocontainers, i.e., in cancer therapy.

## Figures and Tables

**Table 1:** Conditions for experimental reactions for the synthesis of carbon nanotubes (literature).

Methods	Short Description of the Synthesis Conditions	Temperature	Reference
PECVD	Pressure of 5–10 torr, Ni catalyst, reagents: methane and ethane	770 K	[49]
PECVD	Reagents methane + hydrogen	790 K	[50]
PECVD	Reaction with alcohol	720 K	[51]
CVD	Reaction with benzene/ferrocene	670/840 K	[52]
CVD	The reaction with methanol, acetylene, and Co, Co/Fe, Ni/Co/Fe/Al_2_O_3_	770 K	[53]
CVD	The reaction of acetylene with NH_3_/H_2_, Fe/Ni/Co	770–820 K	[54,55]
